# Experimental Investigation on the Structural Performance of Single Span Hollow Core Slab under Successive Impact Loading

**DOI:** 10.3390/ma15020599

**Published:** 2022-01-13

**Authors:** Kamal Amin Chebo, Yehya Temsah, Zaher Abou Saleh, Mohamad Darwich, Ziad Hamdan

**Affiliations:** 1 Civil and Environmental Engineering, Beirut Arab University, Beirut 1001, Lebanon; ytemsah@bau.edu.lb (Y.T.); m.darwich@bau.edu.lb (M.D.); 2 Civil and Environmental Engineering, University of Balamad Dubai, Dubai 00000, United Arab Emirates; zaherabou.saleh@fty.uobd.ac.ae; 3 Civil and Environmental Engineering, Lebanese University, Tripoli 1300, Lebanon; ziad.hamdan.2@ul.edu.lb

**Keywords:** precast slab, successive impact load, concrete damages, cracks, dynamic response

## Abstract

In Lebanon and many other countries where structures are vulnerable to impact loads caused by accidental rock falls due to landslides, specifically bridges with hollow core slab, it is mandatory to develop safe and efficient design procedures to design such types of structures to withstand extreme cases of loading. The structural response of concrete members subjected to low velocity high falling weight raised the interest of researchers in the previous years. The effect of impact due to landslide falling rocks on reinforced concrete (RC) slabs has been investigated by many researchers, while very few studied the effect of impact loading on pre-stressed structures, noting that a recent study was conducted at Beirut Arab University which compared the dynamic behavior of reinforced concrete and post-tensioned slabs under impact loading from a 605 kg impactor freely dropped from a height of 20 m. Hollow core slabs are widely used in bridges and precast structures. Thus, studying their behavior due to such hazards becomes inevitable. This study focuses on these types of slabs. For a better understanding of the behavior, a full scale experimental program consists of testing a single span hollow core slab. The specimen has 6000 mm × 1200 mm × 200 mm dimensions with a 100 mm cast in a place topping slab. Successive free fall drops cases from 14 m height will be investigated on the prescribed slab having a span of 6000 m. This series of impacts will be held by hitting the single span hollow core slab at three different locations: center, edge, and near the support. The data from the testing program were used to assess the structural response in terms of experimental observations, maximum impact and inertia forces, structural damage/failure: type and pattern, acceleration response, and structural design recommendations. This research showed that the hollow core slab has a different dynamic behavior compared to the post tensioned and reinforced concrete slabs mentioned in the literature review section.

## 1. Introduction

Lebanon is characterized by its varied terrain (coast, mountains, and valleys). With the increase in population, people moved to live in the mountain region, increasing the percentage of inhabitants there. Lebanon is one of the countries where many landslides are caused because of heavy rain falling in the winter, particularly in mountain areas, resulting in rock falls threatening lives and causing severe damage to the infrastructure and to the residential buildings. Considering the advantages of precast pre-stressed concrete structures, they were introduced in the industry for construction purposes such as covering large spans and speed of erection compared to conventional RC structures. Noting that the most commonly used bridge deck system used in Lebanon mountain areas are made up using precast hollow core units and because of the high probability of natural catastrophes that may highly cause failure for this structures, it is mandatory to study the behavior of hollow core slab under impact loading and finally provide design recommendations for manufactures and structural enhancement for a better performance under such extreme cases of loading. This complex phenomenon drew the attention of many researchers noting that structural engineers do not take into consideration such type of loading cases though the international design codes used.

Numerous studies have been conducted, experimentally and numerically, on the behavior of reinforced concrete and pre-stressed structures when subjected to impact loads.

Reference [1]: The aim of this study was to investigate the behavior of post- tensioned (PT) slabs under impact load. A comparison was made with a traditional (RC) slab having a similar moment capacity. The RC slab was a flat slab with a thickness of 320 mm, while the other two slabs were PT with a thickness of 250 mm. The slabs were subjected to an impact load of 605 kg dropped freely from 20 m. The load was dropped at the center of gravity for the RC and PT1 slabs. On the other hand, the load was dropped at mid-span of the free edge of the second post-tensioned slab PT2. The tests showed the behavior of the PT slabs under dynamic impact load due to the free falling block, and their different behavior from the RC slab, by comparing displacements, impact force, cracks, and damage type. Additionally, the results displayed the effect of impact location on the response of PT slabs through analyzing PT1 and PT2 results.

Reference [2]: This research aimed to study the efficiency of shear reinforcement as rehabilitation techniques for PT “post-tensioned” slabs damaged by falling rocks. Two simply supported PT slabs were considered in this study. Each had a dimension of 6.6 m × 3 m × 0.25 m and was subjected to an impact from a 605 kg reinforced concrete falling block at a height of 20 m. The first slab (PT-1) was hit at its center of gravity, while the second one (PT-2) was hit at the mid-span of its free edge. After impact, both slabs were repaired by replacing the damaged parts and adding shear ties in order to prevent any future collapse when new impact occurred. The impact test was repeated again after repairing, and both punching shear capacity and normal stresses were recorded. Results showed that the repaired slabs were able to resist the repeated impact successfully. Both punching shear and normal stress capacities were higher than the applied stresses. Moreover, using shear reinforcement helped in changing the crack pattern from shear to flexure. At the end of this study, some recommendations are suggested for further studies.

Reference [3] studied the impact load effect on reinforced concrete road sheds, both experimentally and numerically. The considered reinforced concrete sample of 12 m × 4.4 m × 0.28 m, simply supported over two lines of 11 steel cylinders, were subjected to an impact load from 450 kg reinforced concrete cube. Three impacts were carried out: two of them were from a height of 15 m and one from 30 m. The numerical modelling results using ABAQUS proved to be in accordance with the experimental measurements, where the deflection values measured in the three impacts were approximately equal to the calculated ones. The results showed that the modelling technique used simulated results very close to the experimental ones.

Reference [4] studied six RC slabs and compared the effect of transverse reinforcement or pre- stressing in resisting a missile’s impact. It was concluded that for the same thickness both the transverse reinforcement and the pre-stressing decrease the slab deflection. Yet, pre-stressing tends to decrease the scabbing area and the punching cone angle.

References [5,6,7,8] numerically studied the effect of blast loads on reinforced concrete beams. The studies showed that blast loads caused local damage in the studied beams, and a spalling phenomenon was realized especially in the tensile zone. The slab system was idealized as a single degree of freedom and analyzed to confirm the numerical analysis results, and the damage curves for the studied beams were plotted for different damage levels. The results of the blast phenomenon were similar to those of the falling load impact.

The current study intended to extend understanding and to get a deep insight into the structural response of pre-stressed slabs’ behavior, namely the hollow core slabs when subjected to low-velocity successive impacts.

## 2. Methodology

### 2.1. Geometry and Detailing

The hollow core slab unit was selected based on the thickness to span and characteristic service loads where a 200 mm hollow core slab with a 6 m long span can support 10.5 KPa according to the manufacturer.

### 2.2. Slabs Preparation

The experimental test in this paper is part of an ongoing doctoral program at the department of structural engineering in Beirut Arab University, studying the structural response of concrete structures under impact loading.

- Tested Specimen

**Figure 1:**
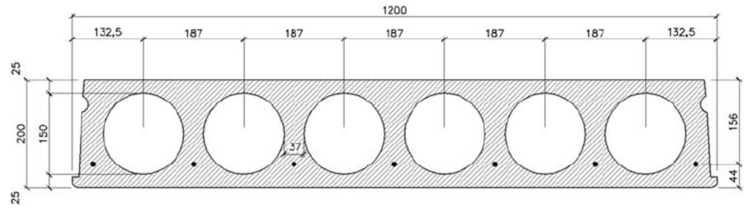
Layout and cross-section details of a typical pre-stressed concrete hollow core slab unit.

The analyzed slab is a structure 6000 mm in length by 2400 mm (width) and 200 mm thickness as illustrated is Figure 1.

**Figure 2:**
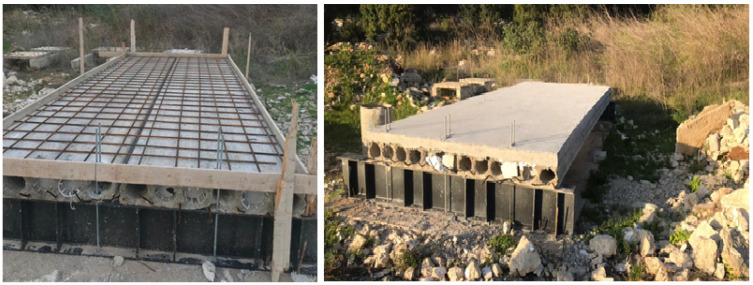
Single span hollow core slab with concrete topping site execution.

The slab system consists of two precast hollow core units. As illustrated in Figure 2 they were placed side by side to accomplish a width of 2400 mm, in addition to a 100 mm cast in place concrete topping.

**Figure 3:**
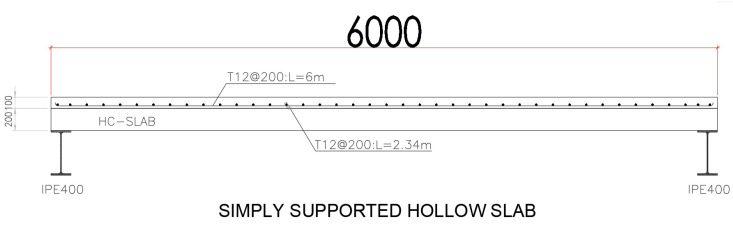
Layout and cross-section details of hollow core with concrete topping.

The topping slab is made of concrete with a 45 MPa compressive strength and includes steel reinforcement bars as illustrated in Figure 3.

Construction materials properties were grouped in Table 1.

The bars are of 12 mm diameter spaced at 200 mm in both directions with no use of shear connectors or any other type of shear reinforcement. They have an average modulus of elasticity and tensile strength of 200 GPA and 515 MPa, respectively.

- Drop Weight

**Figure 4:**
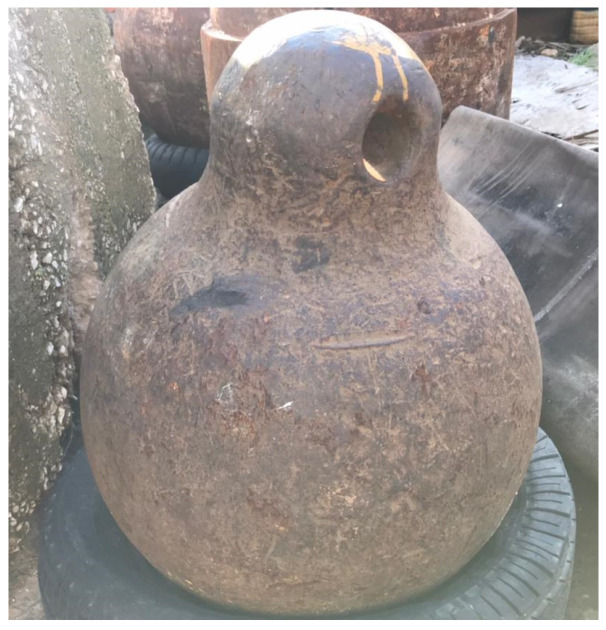
The slab Impactor used: 600 kg Steel Ball.

The mass of the drop-weight used in the experimental program is 600 kg (Figure 4). The drop-weight is a steel ball to ensure that the striking surface of the drop-weight is consistent and to avoid the inconvenience that can take place in case of cube striker with sharp edges and corners.

The impact loads were generated by what was essentially a free-fall condition of the drop weight. The cable of the moving crane attached to the steel ball ran through the center of the drop-weight and was used to guide the weight during the fall.

### 2.3. Test Setup

**Figure 5:**
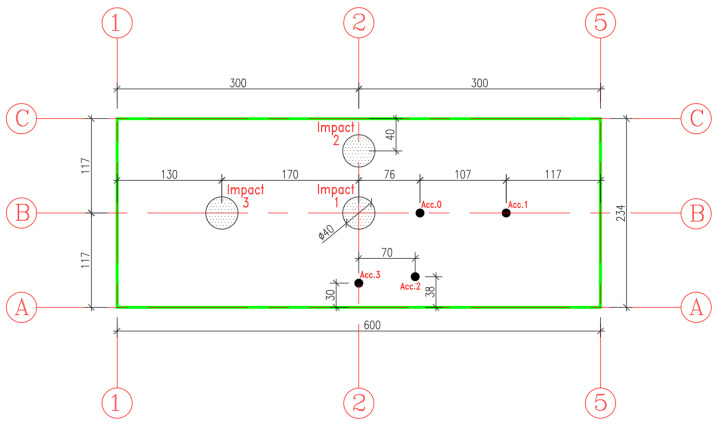
Successive impact locations, layout, and positioning of accelerometers.

The hollow core slab was tested under successive impact loading at three different locations: Slab center, edge, and near the support as illustrated in Figure 5.

**Figure 6:**
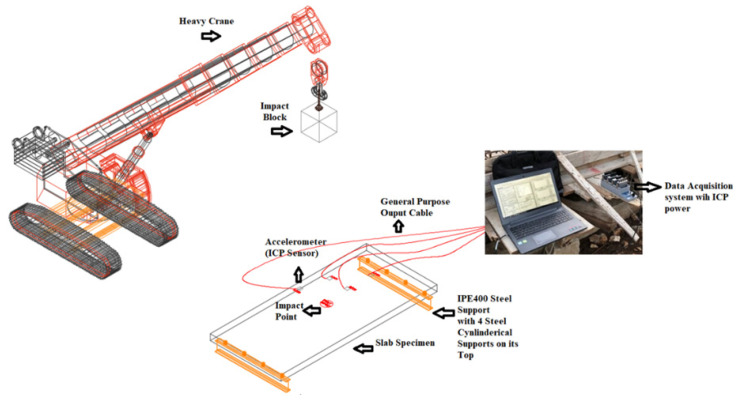
Test setup with all experimental components.

The hollow core slab was mounted by four electronic accelerometer fixed at different positions as shown in Figure 6. The choice of accelerometers’ locations is selected to be excluded from the impacted area to avoid any damage in this sensors which may generate nonrealistic readings. These sensors were attached to a data acquisition system to register readings during the impact

### 2.4. Testing Procedure

The experimental analysis consists of simulating a slab subject to a series of successive impacts. The tests involve dropping a steel ball of 600 kg as illustrated in Figure 5 from a given height of 14 m. The steel ball velocity just before impact is about 16.57 m/s. The impact energy (E) and block velocity (v) just before impact are approximated by mean of the relation:

(1) $$E=M*g*h=\frac{1}{2}{*M*v}^{2}\mathrm{with}\;v=\sqrt{2*g*h}$$

where M, g, and h are the mass of steel ball, gravitational acceleration, and the height of the free fall, respectively.

**Figure 7:**
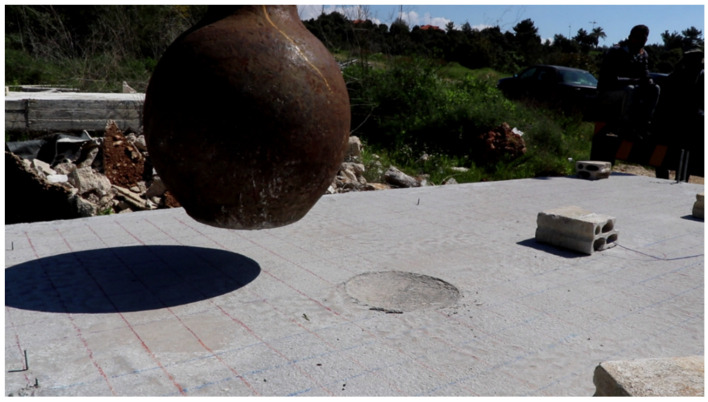
Structural damage in the concrete topping from the first impact.

The drop weight is lifted to the dropping height by a crane as shown in Figure 7. A specific device is used to release the impact weight without any initial speed.

The generated accelerations with respect to time were recorded and the data were saved through the data acquisition system. Then these values were used to derive the displacements and calculating the impact force, as discussed in the following section. The structural assessment of the specimen response to the impacts such as damaged zones, failure pattern, cracks detections, and propagation were registered after each impact, and results were then analyzed.

## 3. Results

Several measurements have been made on the analyzed slab. We present in this paper the results related to the (i) damage analysis, (ii) dynamic behavior in terms of acceleration response, and damping ratio, (iii) shear strength, inertia force, and impact force grouped in Table 2.

### 3.1. Damage Analysis

**Figure 8:**
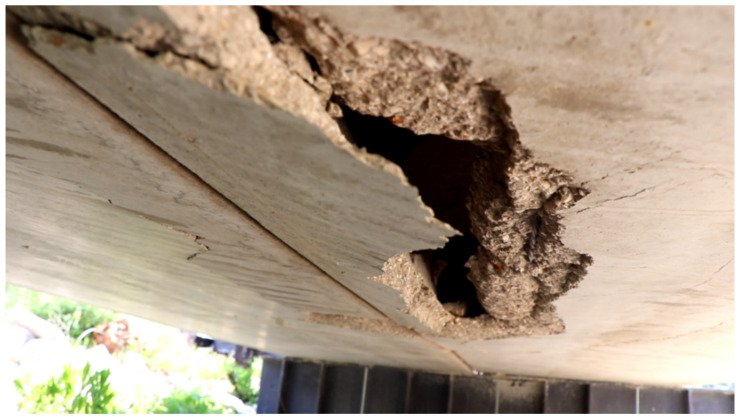
Closed view for the structural damage in hollow core slab from the first impact.

**Figure 9:**
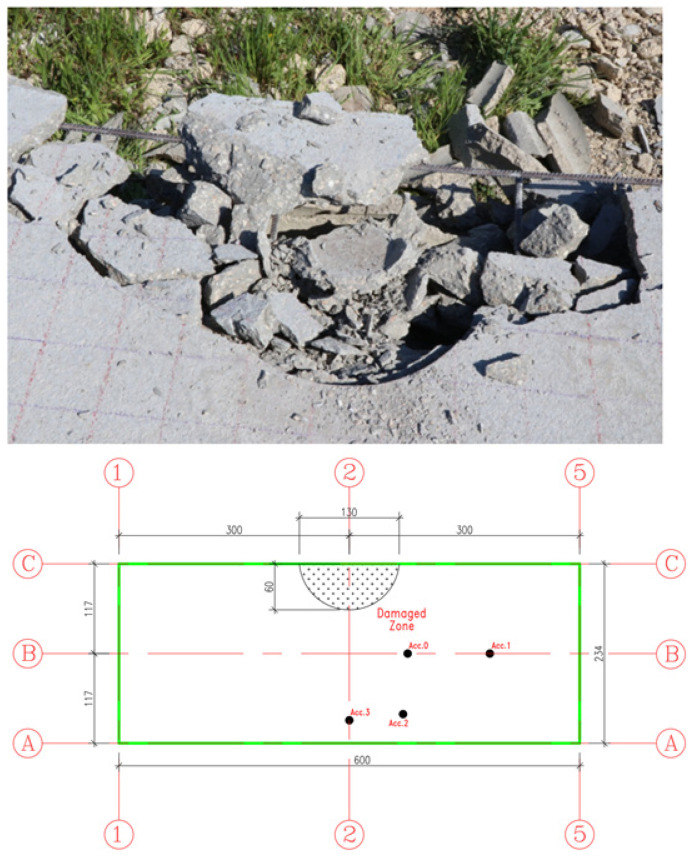
The damaged zone in concrete topping after the second impact.

**Figure 10:**
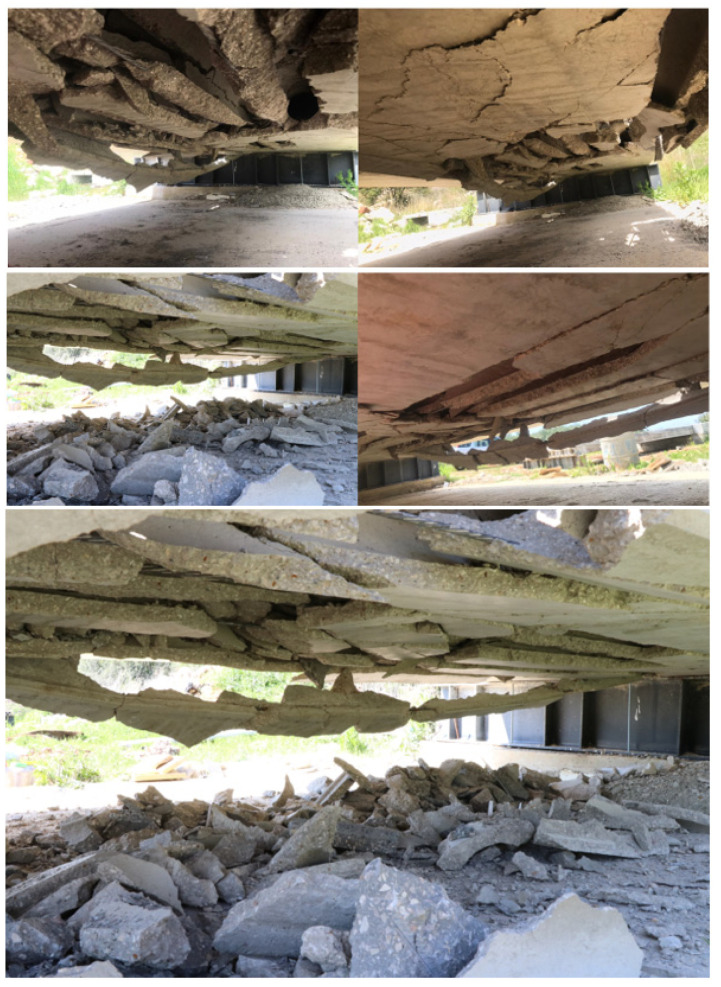
Structural damage from the bottom side after the third impact.

The structural damage in the concrete topping and in the hollow core slab was assessed as following:

- First Impact Experimental Observations (Figure 7 and Figure 8):
  Concrete topping slab damage assessment:
    - Impact crater diameter 28 cm;
    - No cracks in the topping slab;
    - No punching with a 2 cm impact penetration through the slab thickness.
  Hollow core slab damage assessment:
    - No global failure in the hollow core units;
    - Local damage directly beneath the impact represented by the spalling of the 25 mm concrete cover from the bottom side as illustrated in the figure below;
    - Overall damages were concentrated in the lower unreinforced flange of the hollow core unit;
    - No damage in the upper flange of the hollow core unit was assessed (Topping slab and hollow core unit worked compositely);
    - There is no separation between the concrete topping and the hollow core unit;
    - No de-bonding between the embedded strands and the concrete host.
- Second Impact Experimental Observations (Figure 9):
  Concrete topping slab damage assessment:
    - Concrete fracture at the impact location with damaged zone;
    - Transversal crack passing through the depth was initiated in the topping slab and the hollow core unit;
  Hollow core slab damage assessment:
    - Severe shear damage normal to the slab caused a cut off for the first three cores of the hollow core unit;
    - Total concrete facture of the web, top, and bottom flanges of the hollow core unit;
    - Normal and transversal contact separation between the concrete topping and the hollow core units;
    - The first three tendons were de-bonded from the hollow core unit because of the concrete facture of the web, top, and bottom flanges of the hollow core;
    - The total stiffness of the hollow core unit was highly reduced by the loss of three of its cores with their strands;
    - The composite behavior between the concrete topping and the hollow core unit in terms of flexural rigidity was highly reduced because of the surface contact separation;
    - As expected, the edge impact caused a much higher damage in both the concrete topping and hollow core unit as compared to the one done by the first impact at the center.
- Third Impact Experimental Observations (Figure 10):
  Concrete topping slab damage assessment:
    - Impact crater diameter 35 cm;
    - No cracks in the topping slab;
    - Concrete topping was punched through its depth (high shear force near the support);
  Hollow core slab damage assessment:
    - Large damage distributed along the whole parts of the hollow core units;
    - Major concrete facture of the web, top and bottom flanges of the hollow core unit;
    - The brittle damage in the top and bottom thin flanges connecting the webs leads to a total loss of hollow core slab stiffness;
    - The brittle damage in the web connecting the two flanges leads to a total loss of hollow core slab stiffness mainly when the lower part where the pre-stressing strands are embedded separates from the gross the section of the slab;
    - The brittle failure of the top and bottom thin flanges governs the global behavior of the slab.

### 3.2. Dynamic Behavior

**Figure 11:**
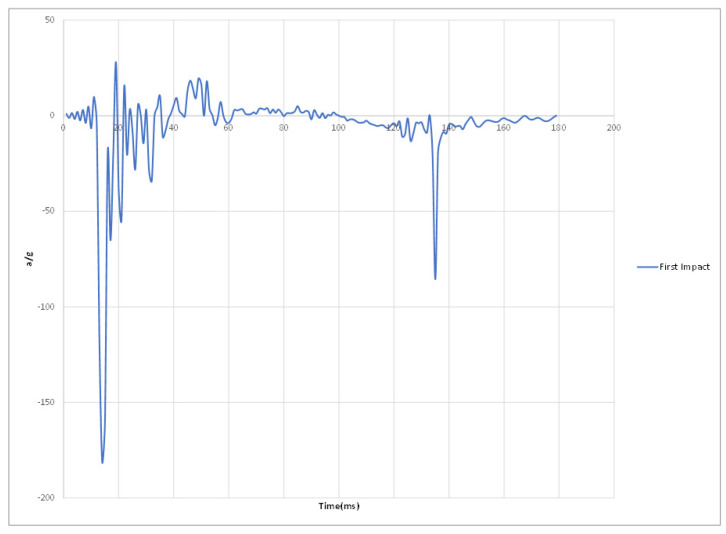
First impact peak accelerations response plot.

**Figure 12:**
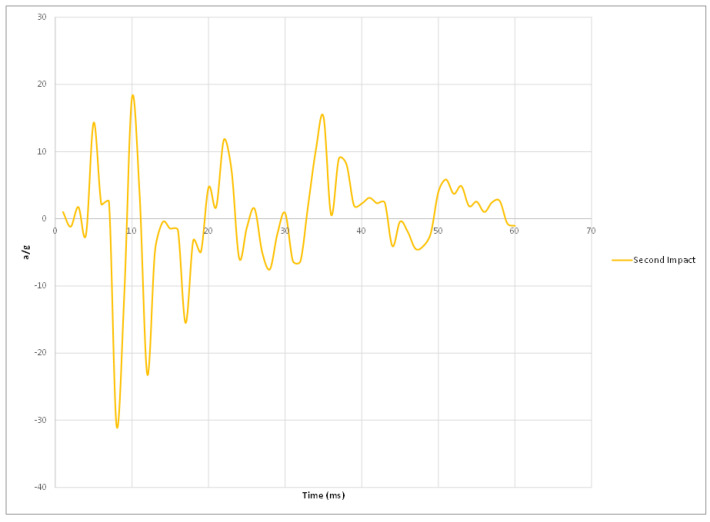
Second impact peak accelerations response plot.

**Figure 13:**
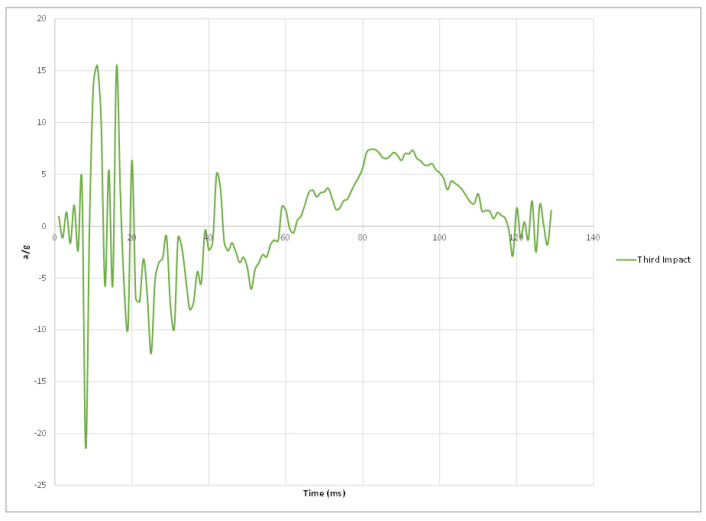
Third impact peak accelerations response plot.

**Figure 14:**
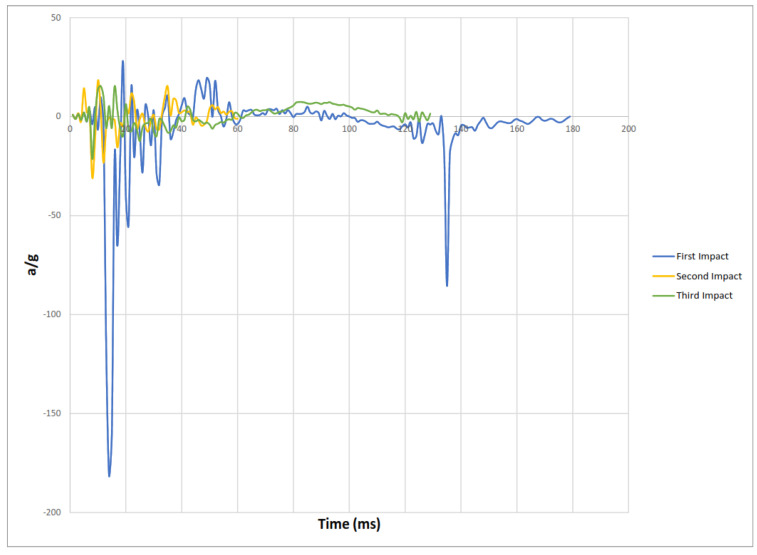
Peak accelerations response plots.

Experimental results of the three impacts are summarized in Table 2 where peak acceleration for each impact was extracted from the accelerometer readings illustrated in Figure 11, Figure 12, Figure 13 and Figure 14.

Figure 14 Peak accelerations response plots.

Damping ratio was calculated by the use of the first two peak accelerations and according to the Equation (3):

(2) $$\delta =\ln\frac{a_{1}}{a_{2}}=\frac{2*\pi }{{\left(1-Ϛ\right)}^{0.5}}$$

(3) $$Ϛ={\left(\frac{\delta^{2}}{4\pi^{2}+\delta^{2}}\right)}^{0.5}$$

Equation (4) from the PCI code was used to calculated the shear strength of the composite slab and found to be equal to 121.27 KN.

(4) $${\varphi *V}_{n}=\varphi *2{\left(f_{c}^{\prime }\right)}^{0.5}\;{*\;b}_{w}\;*d\;+\;0.17{\left(f_{c}^{\prime }\right)}^{0.5}{*\;b}_{0}\;*\;d$$

And Equations (5) and (6) were used to calculate the maximum impact force and the dynamic magnification factor respectively for each impact.

(5) $$\mathrm{Impact}\;\mathrm{force}=\frac{m*g*h}{\Delta }$$

(6) $$\mathrm{DMF}=\frac{\mathrm{Impact}\;\mathrm{Force}}{{\varphi *V}_{n}}$$

## 4. Conclusions

This paper studied the behavior of single span hollow core slab under successive impact load at three different locations: center, edge, and near the support under a 600 kg free falling steel ball from a height of 14 m. The structural response of the slab in terms of damage assessment, acceleration response, damping, and impact force has been studied. The results thus obtained were mentioned and the following conclusions were drawn:

- Structural damages in hollow core units cannot be repaired or strengthened.
- Voids, thin web, and thin flanges are considered the weak points of the hollow core slab.
- The presence of strands has no participation in the section load resistance if the continuity of the webs is lost by the facture of one or both flanges.
- The slab system (HC + Topping) showed a lower capacity in terms of damage and acceleration response for impact at the edge compared to the impact at the slab center.
- The decaying function of the acceleration response represented by the accelerometers readings indicate an unconventional damped vibration. This response can be explained by the following reasons:
  - The severe damage in the slab body led to a vibration with a low acceleration amplitude directly after the impact;
  - The concrete topping as solid section and the hollow core units as voided section vibrate in a different manner under impact loading;
  - The concrete topping and the hollow core units vibrate independently from each other under vertical excitation because of the absence of any type of connectors enforcing the total thickness to work as one unit;
  - The reduction in slab capacity to resist impact can be demonstrated by the ratio of the maximum acceleration response between the first and the third impact $\frac{182*g}{21*g}=8.667$.
- The reinforced concrete topping with high compressive strength (45 Mpa) helped the hollow core slab section to carry more impacts.
- Concrete solid section behaves in a much better way than the hollow section in terms of structural damage and cracks generation.
- Adding shear connectors can enhance the structural response of the slab system (Topping + HC unit) to avoid the contact separation specially in the case of impact at the slab edge.
- Code limitations must be provided to select the minimum flange and web thicknesses in addition to the maximum void diameter in hollow core units.
- Filling material such as foam can be used to absorb a part of the energy induced in the body of the hollow core unit to mitigate the brittle fracture of the thin flanges, therefore enhancing the structural performance of the slab system.
- The presented damping ratio showed the vulnerability of the impact load location in the hollow core slab.
- The reduction in slab impact capacity after successive impacts can be measured by the ratio of impact resistance factor = F1/(F2) = 13,868/1600 = 8.67.

## Figures and Tables

**Table 1:** Material Properties.

Item	HC Slab	Toping Slab
Compressive Strength (MPa)	48	48
Reinforcement	6 ϕ 9.53 1 ϕ 12.5	6 ϕ 12/m
Reinforcement Tensile Strength (MPa)	1860	420

**Table 2:** Summary of experimental results.

Impact	Peak Acceleration	Damping Ratio (%)	Impact Force (KN)	DMF
First Impact	−182 g	16.1	1498.25	12.35
Second Impact	−31 g	5	1008.61	8.317
Third Impact	−21 g	8.5	1275.6	10.52

## Data Availability

The data presented in this study are available on request from the corresponding author.
